# Equity in global health research: A proposal to adopt author reflexivity statements

**DOI:** 10.1371/journal.pgph.0000160

**Published:** 2022-03-30

**Authors:** Sepeedeh Saleh, Refiloe Masekela, Eva Heinz, Seye Abimbola, on behalf of the Equitable Authorship Consensus Statement Group, Ben Morton, Andre Vercueil, Lisa Reimer, Chisomo Kalinga, Maaike Seekles, Bruce Biccard, Jeremiah Chakaya, Angela Obasi, Ndekya Oriyo

**Affiliations:** 1 Department of Clinical Sciences, Liverpool School of Tropical Medicine, Liverpool, United Kingdom; 2 Head of Department of Paediatrics and Child Health, School of Clinical Medicine, College of Health Sciences, University of Kwa-Zulu Natal, Durban, South Africa; 3 Departments of Clinical Sciences and of Vector Biology, Liverpool School of Tropical Medicine, Liverpool, United Kingdom; 4 School of Public Health, University of Sydney, Sydney, Australia; 5 Department of Clinical Sciences, Liverpool School of Tropical Medicine, Liverpool, United Kingdom; 6 King’s College Hospital NHS Foundation Trust, London, United Kingdom; 7 Department of Vector Biology, Liverpool School of Tropical Medicine, Liverpool, United Kingdom; 8 Department of Social Anthropology, University of Edinburgh, Edinburgh, United Kingdom; 9 Department of International Public Health, Liverpool School of Tropical Medicine, Liverpool, United Kingdom; 10 Department of Anaesthesia and Peri-operative Medicine, Groote Schuur Hospital and University of Cape Town, Cape Town, South Africa; 11 Global Respiratory Health, Department of Clinical Sciences, Liverpool School of Tropical Medicine, Liverpool, United Kingdom; 12 Department of Medicine, Dermatology and Therapeutics, School of Medicine, Kenyatta University, Nairobi, Kenya; 13 Department of International Public Health, Liverpool School of Tropical Medicine, Liverpool, United Kingdom; 14 AXESS Clinic, Royal Liverpool University Hospitals NHS Foundation Trust, Liverpool, United Kingdom; 15 National Institute for Medical Research, Dar es Salaam, Tanzania; PLOS: Public Library of Science, UNITED STATES

Parachute (or helicopter) research is a term used for research based in a host country but conducted by external researchers, usually from high-income countries (HICs), with little or no local engagement or appropriate acknowledgement of the local staff, populations, data or infrastructure on which such research relies [1]. Under-representation of authors from Low- and Middle-Income Countries (LMICs) in research based in LMICs has been widely recognised and discussed [2] but little progress has so far been made. A recent analysis found that one fifth of all articles relating to COVID-19 in Africa were published without a local author [3]. In this opinion article, we will make a case for all stakeholders in the research ecosystem (especially academic journals) to adopt a practice in which author reflexivity statements accompany all papers published from HIC-LMIC research partnerships.

Imbalance in HIC-LMIC research partnerships reflects inequities in power and influence inherent in the research ecosystem, where financial limitations in LMICs often lead to inequitable collaborations with HIC researchers. Driven by this power imbalance, inequitable partnerships can further amplify differentials in research skills, knowledge, or experience. In many LMIC settings, limited research infrastructure and few established local senior researchers can mean a shortage of in-country training opportunities, limiting the influence of local collaborators within partnerships. Financial constraints can make it difficult for more junior LMIC partners and women to pursue long-term research career development opportunities, and power differentials can restrict the influence of LMIC partners in agenda-setting and research conceptualisation. Against this background, inadequate recognition of research contribution in terms of authorship further perpetuates systemic inequities [4–6].

Academic journals have an important role in shaping research prioritisation, funding, and norms. Journals can therefore impact on global North-South research inequities; especially as authorship on publications is one of the most valued currencies in academia. As well as assessing the quality of submitted research, journal editors have a responsibility to promote equity and integrity in the research and publication process. This includes reflection on equitable authorship practices, and on global North-South research partnerships in general. Whilst the need for change in practice has been repeatedly discussed, explicit guidance for authors on reporting fair contributorship and for editorial teams to assess equity of partnerships producing research for publication has thus far been lacking. This may be because the editorial teams of academic journals are typically lacking in diversity, and the issue of equity in research, publishing, and authorship have not been perceived to be a problem [7–10].

In January 20201, the publisher *Cell Press* announced a pilot exercise on inclusion and diversity [11]. The corresponding author of research papers in *Cell Press* journals are now required to (on behalf of other authors) complete an inclusion and diversity questionnaire at the point of acceptance. Authors may choose to publish such an Inclusion and Diversity statement alongside their paper. Authors may also opt out of completing the questionnaire. More recently, *PLOS* announced a similar policy on inclusion in global research, to be implemented by all *PLOS* journals [12]. Authors conducting global research may be asked to complete a questionnaire that outlines ethical, cultural, and scientific considerations specific to inclusivity in global research. The questionnaire also asks authors conducting research without local authors why none have been included on the authorship list. While for both initiatives, completing the questionnaire is either optional (*Cell Press*) or by editors’ invitation (*PLOS*), the policies mark a promising shift towards taking equity issues more seriously within the research ecosystem.

In a recent consensus statement, we–a group of journal editors (of journals in fields ranging from basic sciences to clinical medicine, and to public/global health) and researchers (from and based in LMICs)–proposed the use of structured reflexivity statements, to be submitted by authors and published alongside manuscripts, as a mechanism for the standardised, comprehensive assessment of transnational research partnerships, conduct and reporting [13]. We outlined a specific list of considerations for authors to address in their structured reflexivity statements, including the origin of the research question and study design, support for local capacity, how authorship was assigned, especially in relation to gender balance, early career researchers and recognition of local leadership. A suggested assessment checklist is also provided to support editors in evaluating these statements. Finally, we made additional recommendations for journals and editors, including removal of arbitrary authorship limits, expectation of fair acknowledgement for local authors, support of LMIC research capacity, and aim to address wider structural imbalances in research practice and reporting. We suggest these recommendations be considered more widely across the academic publishing system.

We specifically call on journals that publish international work to adopt these statements by requiring that manuscripts submitted from global North-South partnerships should include such structured author reflexivity statements which should then be included in the overall assessment of manuscript suitability for publication. We call on research organisations or relevant units of universities, think tanks, NGOs, humanitarian organisations, United Nations agencies–to adopt the statement so that research submitted by their employee will include an appendix with the structured reflexivity statements even if a journal does not require it. We also call on funders to include such a requirement for international research that they support to promote equitable international North-South research partnerships. Finally, we call on individual researchers to adopt the inclusion of structured author reflexivity statements as an appendix to manuscript submissions from international North-South research partnerships, even when a journal does not require it.

Like completing a declaration of competing interests, our aspiration is that adopting this reflexivity statement will over time encourage researchers to proactively consider equity of partnerships from research conceptualisation, thus promoting fairer practices and addressing parachute research and other questionable research practices. Examples of good practice published in reflexivity statements will pave the way for novel, more equitable ways of conducting collaborative research. Lessons from its implementation by journals currently signing up to introduce the author reflexivity statements, along with experiences from publishers like *Cell Press* and *PLOS*, will also help to improve its proposed structure and content. Collectively, these initiatives should encourage journals and other actors in the research ecosystem–including funders, employers, and individual researchers–to actively address parachute research and other unfair research practices, by introducing mechanisms to monitor, manage, and improve the conduct of research collaborations in situations of power imbalance.
